# Controlled Human Infection to Speed Up SARS-CoV-2 Vaccine Development

**DOI:** 10.3389/fimmu.2021.658783

**Published:** 2021-03-12

**Authors:** Marc Baay, Pieter Neels

**Affiliations:** ^1^ P95 Epidemiology & Pharmacovigilance, Leuven, Belgium; ^2^ International Alliance for Biological Standardization—IABS, Geneva, Switzerland

**Keywords:** coronavirus, vaccine development, controlled human infection (CHI), human challenge, ethics

Controlled human infection (CHI) studies have been performed previously with a range of virologic, bacteriologic, and parasitic agents, yielding important information on immunopathogenesis, duration of vaccine-induced immunity, and to define correlates of protection in healthy populations (1–3). CHI can also provide key safety, tolerability, immunogenicity, and efficacy data supporting vaccine research. Typically, in a CHI experiment, participants are given a test vaccine and are inoculated 2–4 weeks later with the pathogen of interest *via* the most physiologically relevant route of administration. Such experiments require only a few months to complete, and the number of participants enrolled is usually rather in the tens than in the hundreds, let alone in the thousands or ten-thousands needed for phase 3 Randomized Controlled Trials (RCTs).

The COVID-19 pandemic has a major impact on global health. Currently (February 2021), more than 110 million people have (had) an infection with SARS-CoV-2, and nearly 2.5 million died of COVID-19.^1^ Large parts of the world are in the second wave, with no view of imminent solutions, although vaccine development is continuing at unprecedented speed. A number of clinical trials are already ongoing with 73 candidates, including 16 candidates in phase 3 (4). Although the Pfizer/BioNTech, Moderna and AstraZeneca vaccines have been fully evaluated and licensed for emergency use, a second wave of vaccines is under development and worthwhile to become available, given the global market need to cover immunization of seven billion people. If some of the vaccines need two doses to complete primovaccination, that brings the global need quickly to 10 billion doses or more. Today, we cannot say if this will be a yearly need, since duration of immunity will only be known later on. In addition, it cannot be excluded that mutations require quick adaptation of vaccines to potentially new strains. To mitigate the risks of efficacy and availability, we will have to rely on a range of vaccines.

Admittedly, RNA vaccines, nanoparticle vaccines, and virus like particle vaccines (5, 6) can be produced substantially faster compared to conventional expression systems (7), under the assumption that manufacturing facilities are up and running, with sufficient supplies and staffing. For example, in the case of inactivated or live-attenuated viral (such as virus vaccine candidates being produced in cell lines), or recombinant protein vaccine candidate production, product-specific manufacturing processes have to be developed and ideally optimized, validated, and approved for cGMP production, which can take a substantial amount of time. Under ideal circumstances, one billion doses of mRNA vaccine can be produced per year, and nanoparticle vaccines as well as virus-like particle vaccines can be upscaled quickly (5), indicating platform technologies will be of utmost importance to respond to global need of COVID vaccines.

Furthermore, the stability of a vaccine can also influence its global distribution and availability. Vaccines which require distribution and storage at (ultra)low temperatures (−20 to −70°C), would limit distribution in low- and middle-income countries due to the lack of appropriate cold chain infrastructure and would limit the use of multidose vials. The global role out of vaccination would be stimulated by single dose vaccines and having distribution systems already in place (GAVI, the Vaccine Alliance, UNICEF, existing cold chain). Hence, we will have to rely not only on more vaccines but also on diverse vaccines. Until those vaccines are available, social distancing and face masks are the only options, which have a profound impact on global economy, as well as mental wellbeing.

The role of vaccines in limiting circulation of wild virus is under discussion. Many studies in animal models show interesting potential of vaccines to clinically protect and reduce the circulation of virus after subsequent infection (8–13). Those results show the potential impact of different vaccines on the reduction of virus circulation in the population. One could object that extrapolation of results obtained in animals, including non-human primates, are difficult to extrapolate to humans. This might be one of the reasons to consider CHI.

A SARS-CoV-2 CHI model will not be able to provide all answers but is potentially valuable to select between the large number of vaccines and adjuvants in development. This alone would accelerate the development of vaccines as it prevents wasting time and money on large phase II/III studies of ultimately unsuccessful vaccine candidates. Furthermore, in specific situations, especially between COVID-19 waves with low circulating virus, a SARS-CoV-2 CHI model has the potential to provide more rapid solutions to this global problem. Similarly, once safe and effective vaccines have been approved and are being rolled-out, it will become more complicated to perform large-scale RCTs with placebo arms, as the use of placebo arms may not be considered ethical if adequate vaccines are available. Small-scale CHI studies may quickly show the efficacy of the vaccine, potentially with multiple study arms *versus* a single placebo arm, to limit that group to a minimum.

Several other CHI models have been developed and used to support vaccine development. The best-known example is the cholera model: it has been used for proof of concept of Dukoral™, and more recently a challenge model was used for the efficacy part of the evaluation of Vaxchora™. The US-FDA has licensed Vaxchora based on the data of this challenge trial (14). Other mature models have been developed for viruses, bacteria, and parasites, including norovirus, RSV, *Salmonella typhi*, *Streptococcus*, *Shigella*, malaria, and schistosomiasis (15–21).

Thus, despite having three vaccines approved, more valuable candidates need to be selected. A SARS-CoV-2 virus, which can be used as a challenge agent, needs to be developed. The quality of challenge material can be assured by building in safety, using well established quality principles and practices (22, 23).

In parallel, a SARS-CoV-2 CHI model needs to be developed. The ethics of a CHI model for SARS-CoV-2 has been extensively discussed (24–30). CHI models carry inherent risks: participants may develop severe disease or complications after deliberate infection; prolonged isolation may negatively impact their wellbeing; the experimental virus strain may cause a community outbreak. However, with proper risk mitigation strategies, risks can be minimized (31). Initially, the inclusion criteria for CHI studies can be limited to subjects at the lowest possible risk, *i.e.*, 20–30-year-olds. In these challenge trials the infectious dose can be determined, starting from a very low dose. With this experience, experiments in older age groups may become possible, which would be very valuable, as this age group is the major target population for the vaccines, and the data obtained with the younger age groups may not be extrapolated to this older age group (22).

While the risk in the lowest age group is low, it is not non-existent. One year into the pandemic, we still do not have a rescue treatment, which would make CHI acceptable without further ado. Hence, study subjects will have to be observed rigorously, in quarantine units, to be able to do whatever possible to avoid extremely serious consequences, at the earliest possible stage.

Actions towards the development of a SARS-CoV-2 CHI model would represent a broad and sustained research effort toward understanding coronavirus biology and mitigating the current and potentially also future pandemics. There is also a push from civil society; over 38, 000 people have signaled their willingness to participate in CHI studies with 1Day Sooner, a non-profit organization advocating for these volunteers (32).

## Author Contributions

All authors contributed to the article and approved the submitted version.

## Conflict of Interest

The authors declare that the research was conducted in the absence of any commercial or financial relationships that could be construed as a potential conflict of interest.

## Footnotes

1 Johns Hopkins; https://gisanddata.maps.arcgis.com/apps/opsdashboard/index.html#/bda7594740fd40299423467b48e9ecf6
